# Hantavirus Gn and Gc Envelope Glycoproteins: Key Structural Units for Virus Cell Entry and Virus Assembly

**DOI:** 10.3390/v6041801

**Published:** 2014-04-21

**Authors:** Nicolás Cifuentes-Muñoz, Natalia Salazar-Quiroz, Nicole D. Tischler

**Affiliations:** 1 Molecular Virology Laboratory, Fundación Ciencia & Vida, Av. Zañartu 1482, Ñuñoa, Santiago 7780272, Chile; E-Mails: nic.cifuentes@gmail.com (N.C.-M.); salazarqn@gmail.com (N.S.-Q.); 2 Facultad de Ciencias Biológicas, Universidad Andrés Bello, Av. República 237, Santiago 8320000, Chile

**Keywords:** hantavirus, envelope, glycoprotein, cell entry, fusion, biogenesis, assembly, budding, structure

## Abstract

In recent years, ultrastructural studies of viral surface spikes from three different genera within the *Bunyaviridae* family have revealed a remarkable diversity in their spike organization. Despite this structural heterogeneity, in every case the spikes seem to be composed of heterodimers formed by Gn and Gc envelope glycoproteins. In this review, current knowledge of the Gn and Gc structures and their functions in virus cell entry and exit is summarized. During virus cell entry, the role of Gn and Gc in receptor binding has not yet been determined. Nevertheless, biochemical studies suggest that the subsequent virus-membrane fusion activity is accomplished by Gc. Further, a class II fusion protein conformation has been predicted for Gc of hantaviruses, and novel crystallographic data confirmed such a fold for the Rift Valley fever virus (RVFV) Gc protein. During virus cell exit, the assembly of different viral components seems to be established by interaction of Gn and Gc cytoplasmic tails (CT) with internal viral ribonucleocapsids. Moreover, recent findings show that hantavirus glycoproteins accomplish important roles during virus budding since they self-assemble into virus-like particles. Collectively, these novel insights provide essential information for gaining a more detailed understanding of Gn and Gc functions in the early and late steps of the hantavirus infection cycle.

## 1. Introduction

Hantaviruses are human pathogens that are distributed worldwide and classified by the NIH as category A priority pathogens. These viruses belong to the *Bunyaviridae* family, in which over 300 viruses are grouped into five different genera: *Nairovirus*, *Orthobunyavirus*, *Phlebovirus*, *Tospovirus* and *Hantavirus* [1]. Unlike other viruses from this family, which are transmitted by arthropods, hantaviruses are harbored by small mammals, mainly rodents [2]. Their transmission to humans can cause the development of two severe diseases; in Europe and Asia prototype members of the genus, such as Hantaan virus (HTNV), Puumala virus (PUUV) or Dobrava virus (DOBV), are associated with hemorrhagic fever with renal syndrome (HFRS), causing mortality rates that vary from 0.3% to 10%. In North- and South America, hantaviruses such as Sin Nombre virus (SNV) and Andes virus (ANDV) cause the hantavirus cardiopulmonary syndrome (HPS) with mortality rates that vary between 30%–40% [2,3,4].

As all members of the *Bunyaviridae* family, hantaviruses are characterized by a genome comprising three segments of negative sense ssRNA. These segments are coated with multiple copies of the nucleoprotein and are associated with the RNA-dependent RNA polymerase (RdRp), forming the viral ribonucleocapsids. The three ribonucleocapsids are further enclosed by an envelope composed of a lipid bilayer in which two glycoproteins, Gn and Gc, are anchored [1]. Such a viral envelope projects the glycoproteins as spike associations and is acquired by the budding of viral ribonucleocapsids through a cellular membrane in which the viral glycoproteins are inserted. This step enables enveloped viruses to cross the membranous barriers of cells unnoticed, a process that together with secretion allows the exit of virus particles from infected cells [5,6]. In a similar manner, viral envelopes also permit viruses to enter cells discreetly by receptor attachment and virus-cell membrane fusion. Hence, viral envelopes allow virus disassembly and assembly during cell entry and exit, respectively. At the same time these metastable structures confer resistance to extracellular stress factors. This striking duality is crucial for virus cell-to-cell transmission and dissemination within and among hosts (reviewed in [5,6,7,8,9]).

In this review we summarize the current progress in understanding hantavirus envelope glycoprotein functions and highlight recent insights into glycoprotein structures and their possible arrangements on hantavirus particles.

## 2. Structure of the Hantavirus Glycoproteins

**Figure 1:**
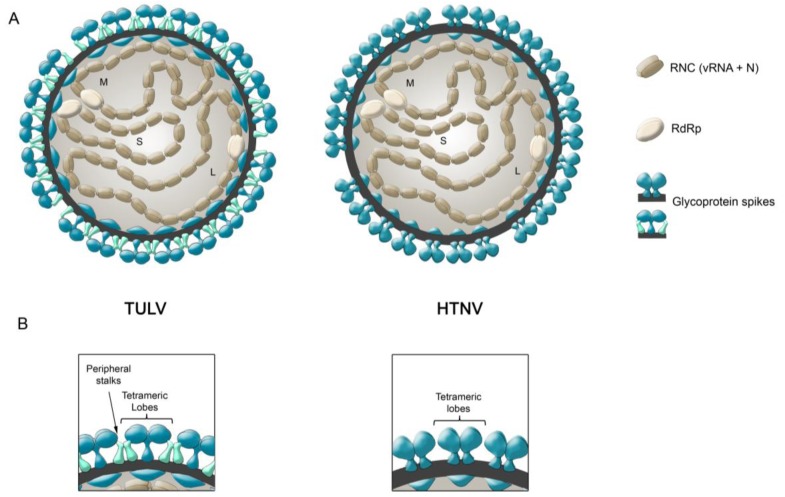
Schematic Representation of Hantavirus Particles. (**A**) Representation of TULV and HTNV particles. Surface spike organizations are based on the cryo-ET maps of each virus. (**B**) Amplified view of the spike arrangements of HTNV and TULV represented in (**A**). Spike projections were drawn based on a vertical slice through the volume of the electron density of cryo-ET maps obtained by Huiskonen and colleagues [17] and Battisti and colleagues [16], respectively. The maps were downloaded from EMDataBank, accession numbers 1704 (TULV) and 2056 (HTNV) and surface projections rendered in the Chimera program [18].

Early electron microscopy (EM) studies described hantaviruses as roughly spherical particles of heterogenic size, varying from 70–160 nm [10,11,12,13,14,15]. More recent cryo-electron microscopy (cryo-EM) studies defined their size range between 120–160 nm and also described the presence of elongated particles of up to 350 nm in length and 80 nm in diameter [16,17]. Yet, it remains to be determined if elongated, pleomorphic structures correspond to technical artifacts or infectious particles. The combination of cryo-electron tomography (cryo-ET) and averaging the tomographic sub-volumes of the Tula virus (TULV) and HTNV at 3.6 nm and 2.5 nm resolution, respectively, has allowed substantial progress in characterizing the ultrastructural surface organization of hantaviruses [16,17]. These studies have revealed that hantaviruses contain square-shaped surface spikes of four-fold symmetry and that each spike protrudes ~12 nm (TULV) and ~10 nm (HTNV) from the viral membrane. Moreover, it has been suggested that the molecular mass of each tetrameric spike complex corresponds to four Gn and four Gc subunits [16,17]. Interestingly, some differences have been observed between the spike densities of HTNV and TULV cryo-ET maps [16,17] which have been depicted in Figure 1. For example, in the case of the TULV map, four membrane associated linkages termed “peripheral stalks” inter-connect adjacent spike complexes. Such peripheral stalks have not been detected in the electron density maps of HTNV. Further, the tetrameric spike complexes of the TULV map are connected to the viral membrane via a central stalk whereas the tetrameric spike complex of the HTNV map contains four stalk densities [16,17]. In the future, it will be interesting to assess if the observed differences between HTNV and TULV spike densities actually exist or if they are caused by technical variations during sample preparation.

Novel ultrastructural analyses of other bunyaviruses are now also available and have revealed an unexpected diversity in terms of their envelope architecture and spike organization. Cryo-EM and single particle reconstruction of the phleboviruses RVFV and Uukuniemi virus (UUKV) indicate an icosahedral T = 12 quasi-symmetry. This highly-ordered arrangement of the surface glycoproteins consists of pentameric and hexameric capsomer associations [19,20,21,22]. It has been also observed that low pH treatment of UUKV induced changes in the viral surface projections from a “tall” (13 nm) to a “flat” (8 nm) appearance [20]. No exposed membrane patches were detected in the architecture of these viruses. For RVFV it has been further suggested that the capsomers are most probably composed of Gn-Gc heterodimers, five to form pentons and six to build hexons [22]. Strikingly, the recent cryo-ET information obtained about the Bunyamwera orthobunyavirus (BUNYV) disclosed an additional different structure, consisting of non-icosahedral particles with glycoprotein spikes projecting in a unique tripod-like arrangement [23]. Each spike is thought to be composed of three Gn-Gc heterodimers. At neutral pH, the spikes project ~18 nm from the virus membrane and similar to phleboviruses, they change their conformation upon low pH treatment [23]. Similar to hantaviruses, exposed membrane patches were also detected on the surface of BUNYV [23]. It is remarkable that despite the structural diversity of the surface projections of bunyaviruses, their surface spike associations seem to be constantly formed by Gn and Gc heterodimeric subunits.

Besides the cryo-ET data of bunyaviruses, little information is available regarding the molecular structure of their glycoproteins. For hantaviruses, the molecular structure of a part of the Gn tail (residues 543–599), has been solved by nuclear magnetic resonance (NMR) spectroscopy [24]. This region revealed the structure of a dual ββα-fold zinc finger that comprises a conserved cysteine/histidine motif, remarkably similar between ANDV and Prospect Hill (PHV) hantaviruses [24,25]. It has been suggested that this region is the only structured part within the hantavirus Gn tail [25]. Despite the low sequence identity between the Gn tail of hantaviruses and other members of the *Bunyaviridae* family, a dual ββα-zinc finger domain has also been described for the Gn tail (residues 729–805) of the Crimean Congo Hemorrhagic fever nairovirus (CCHFV) [26]. Although these zinc fingers have a common global fold, their local folding and electrostatic charge distribution are different. Moreover, recent evidence suggested that these zinc fingers, or adjacent regions, are likely involved in virus assembly [26].

While the molecular structure of the Gn ectodomain has not yet been solved for any member of the *Bunyaviridae* family, a recent article described for the first time the crystal structure of the RVFV Gc ectodomain at 1.9 Å resolution [27]. The overall fold of this structure resembles a class II membrane fusion protein, strengthening previous evidence suggesting that Gc of hantaviruses and other members of the *Bunyaviridae* are fusion proteins arranged into a “class II fold” [28,29,30,31,32]. Class II fusion proteins are composed of three domains formed mainly by β-strands; among them domain I contains the N-terminal of the protein, domain II exposes one or more fusion loops responsible for inserting into target membranes, while the more C-terminally located domain III connects to a flexible linker or so called stem region that binds the ectodomain to a transmembrane anchor [32,33,34,35]. Not all members of the *Bunyaviridae* may bear class II fusion proteins since BUNYV Gc homooligomerization seems to be unlike any currently known fusion protein [23].

## 3. Roles of the Glycoproteins during Virus Cell Entry

**Figure 2:**
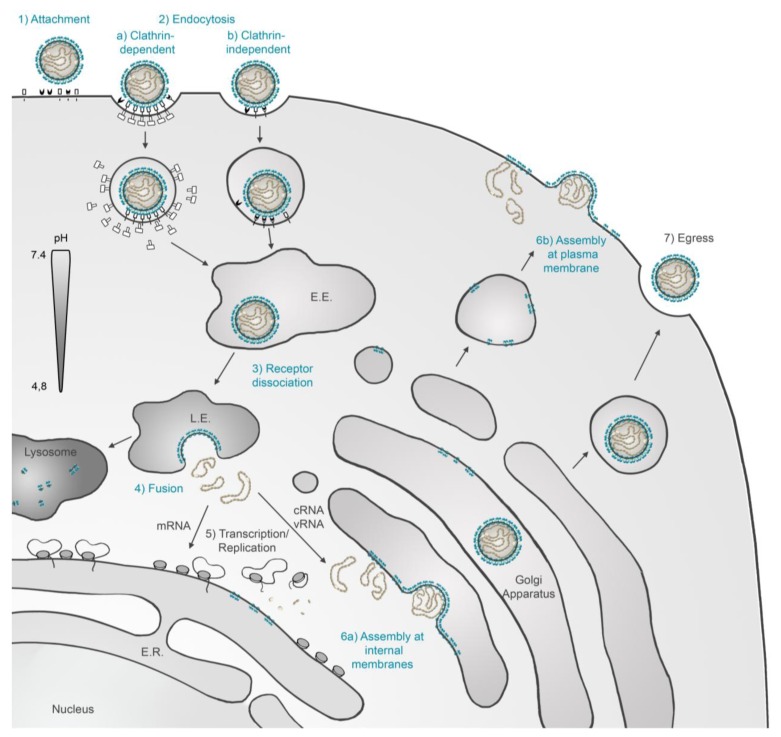
Functions of Hantavirus Envelope Glycoproteins during Virus Life Cycle. Once hantavirus particles attach to cell surface receptors by one, or both glycoproteins (1) they are uptaken by clathrin-mediated endocytosis (2a) or alternatively by other pathways that do not involve clathrin (2b). After virus particle uptake, the viral glycoproteins dissociate at one point from cellular receptors (3). Virus particles traffic down through the endocytic pathway until the low pH of endosomes, and possibly other cellular factors, trigger the Gc-mediated virus-cell membrane fusion process, releasing RNCs to the cytosol (4). After the transcription/replication of the viral genome and the synthesis of new viral proteins have taken place (5), the glycoproteins accumulate at internal membranes, where they can mediate virus assembly and budding (6a) and egress through the secretory pathway (7). Alternatively, new viruses may assemble and bud directly from the plasma membrane (6b). E.E. indicates early endosome, L.E. indicates late endosome.

To enter cells, viruses must pass through the cellular membrane barrier. For this, they have evolved specific entry mechanisms that follow tightly controlled, successive steps [8,36,37]. During this process, at least two key functions must be fulfilled by the hantavirus envelope glycoproteins: (i) interaction with cellular surface receptors and (ii) escape from the endocytic pathway through fusion of viral and cellular membranes. A scheme that summarizes these functions during the virus life cycle is shown in Figure 2.

### 3.1. Interaction of the Glycoproteins with Cellular Receptors

The initial step of the virus-cell entry involves virus binding to the cell surface via attachment factors, or to cellular surface proteins serving as receptors. The latter actively promotes virus entry by triggering conformational changes in the virus, through activation of signaling pathways or by mediating endocytic uptake. In addition, viruses may use several receptors in a consecutive or parallel manner (reviewed in [8,37]). Hence, the interaction of viral envelope glycoproteins with entry receptors also determines the host cell tropism of viruses. Hantaviruses replicate principally in endothelial cells and it has not yet been determined whether the Gn or Gc glycoprotein alone, or both consecutively, interact with cell surface molecules (see Figure 2, step 1). Currently, several cellular proteins have been proposed as hantavirus receptors, including β3 integrins, decay-accelerating factor (DAF)/CD55 and the receptor for the globular head domain of complement C1q (gC1qR)/p32 [38,39,40,41,42]. Early studies identified α_V_β3 integrins as molecules allowing *in vitro* entry of HFRS and HPS-associated hantaviruses [38,39]. Subsequently, the interaction of HTNV particles with β3 integrins and an unknown 70 KDa protein has been described [43]. Moreover, a specific plexin-semaphorin-integrin (PSI) domain within the N-terminal of β3 integrins has been described as the site of interaction with pathogenic hantaviruses [44,45]. In addition, *in vitro* evidence indicates that HTNV and PUUV use the glycophosphatydilinositol (GPI)-anchored DAF to attach to the apical surface of polarized epithelial cells. It has been suggested that this interaction may allow trafficking of HTNV to the basolateral site of polarized cells [41]. Also, a direct interaction between HTNV particles and the gC1qR, has been reported [40,42]. It remains to be studied in future experiments, if some of these receptors are also being used *in vivo* in hantavirus-infected individuals.

The binding of viruses to a receptor through individual contacts can be highly specific, but is often of low affinity (reviewed in [7,37]). However, binding to multiple receptors clustered in membrane microdomains, enhances avidity and can also trigger receptor signaling, favoring the generation of membrane curvature and activating endocytosis. Classical membrane microdomains are enriched in cholesterol [7]. In this context, when cells were depleted of cholesterol, they became less susceptible to infection by hantaviruses and other viruses of the *Bunyaviridae* family [41,46,47]*.* Cholesterol not only acts as a docking site for specific proteins, but also modulates membrane viscosity and may be required for the virus-cell fusion process [48,49,50]. Hence, the exact role that cholesterol may play during hantavirus cell entry has not yet been determined.

### 3.2. Endocytic Uptake and Pathways of Hantaviruses

The cellular pathways that hantaviruses and other bunyaviruses use to enter target cells remain largely uncharacterized. Drug-based clathrin inhibition studies showed that the Black Creek Canal (BCCV), HTNV and Seoul viruses seem to be internalized through clathrin-mediated endocytosis, whilst ANDV may use a mechanism that does not involve clathrin (Figure 2, step 2a,b) [51,52]. Different endocytic requirements have also been described for other bunyaviruses; studies based on the use of inhibitors or siRNA-silencing of clathrin or adapter protein 2 (AP-2) showed that Oropouche and La Crosse (LACV) orthobunyaviruses as well as CCHFV nairovirus internalization is mainly clathrin-dependent. On the other hand, the UUKV phlebovirus endocytic uptake seems to be largely clathrin-independent [46,53,54,55,56].

How far viruses hitchhike on the endocytic pathway before they escape into the cytosol depends mostly on their membrane fusion proteins and the respective pH at which their activation is triggered. An early report indicates that HTNV may co-localize with the early-endosome marker EEA-1 [51]; however the study was conducted 90 min post-infection and hence no clear conclusions can be drawn. A recent study showed that UV-irradiated SNV increased levels of Rab7-activated molecules 15 min post-infection [52]. Further studies are required to shed light on the still poorly characterized endocytic pathway of hantaviruses. For other bunyaviruses, the passage through the endocytic pathway seems to depend also on each virus. The infection of cells expressing dominant-negative Rab5 and Rab7 mutants indicated that LACV and CCHFV traffic only through early endosomes. On the other hand, studies based on EM, fluorescence microscopy and siRNA, showed that UUKV and Oroupouche virus seem to reach late endosomal compartments [46,53,54,55]. In the case of CCHFV, an additional study indicates that intact microtubules are required during early time points, suggesting that this virus may also enter cells through late endosomes [56].

### 3.3. Glycoprotein-Mediated Membrane Fusion

During endocytic uptake and hitchhiking on the endosomal pathway, viruses remain enclosed by endosomal membranes, a barrier that must be crossed in order to avoid their degradation in lysosomal compartments and to instead achieve the release of their ribonucleocapsids into the cytosol. Enveloped viruses face this step by fusing their membranes with the membrane of endosomes. This process is mediated by viral fusion proteins that are anchored at the viral membrane by a transmembrane domain (reviewed in [57,58]). Based on diverse crystallographic and biochemical information, a general virus-cell membrane fusion mechanism consisting of multiple steps has been proposed. First, a trigger induces conformational changes in the fusion protein that leads to the exposition of a fusion peptide-or fusion loop-that inserts into the target membrane, thereby bridging the two opposed membranes. Subsequently, fusion proteins trimerize, and after different conformational changes, this extended intermediate conformation collapses in order to bring the fusion peptide and transmembrane region into close vicinity. This movement also approximates the opposed membranes until they reach hemifusion and the subsequent full fusion of both membranes, thereby opening a fusion pore that allows the delivery of the viral ribonucleocapsids into the cytosol. At this stage, the fusion proteins have reached their post-fusion conformation, a trimeric, hairpin-like structure that in the case of class I and II fusion proteins is irreversible [36,59].

**Figure 3:**
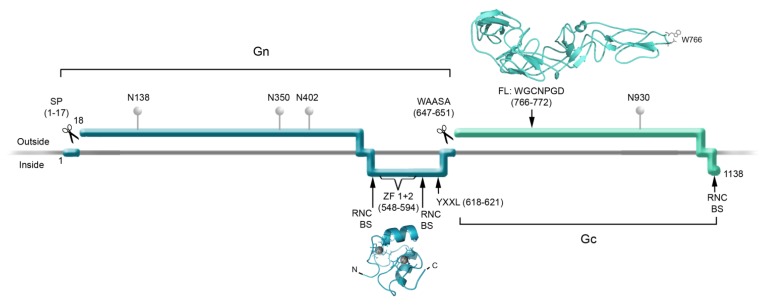
Schematic Representation of Hantavirus Glycoprotein Processing and Functions. The Gn (dark cyan) and Gc (light cyan) glycoprotein ectodomains, transmembrane regions and endodomains are represented according to their location relative to the membrane (horizontal grey line). The Signal peptide (SP) and WAASA sequences indicate the cleavage sites within GPC. N represents the location of asparagine residues that are likely to carry glycosylations; the numbers following these letters indicate the corresponding residue number within GPC of ANDV. ZF 1 + 2 indicates the location of the two zinc finger domains, RNC-BS indicates the suggested ribonucleocapsid binding sites. The motif YxxL represents residues involved in ubiquitination. FL indicates the location of the putative fusion loop of ANDV Gc. The molecular structure of the ANDV Gn-CT zinc finger domains (dark cyan) was obtained from the NCBI databank, PDBid: 2K9H [24]. The molecular model for the ANDV Gc fusion protein ectodomain was developed previously [29]. All ribbon diagramsof molecular structures were rendered in the PyMOL Molecular Graphics System [61].

For hantaviruses, the membrane fusion activity was first associated with the Gn and Gc glycoproteins by Ogino and colleagues [60]. Subsequently, a highly conserved sequence that *in vitro* interacts with liposomes was identified as a fusion peptide candidate within Gc [29]. Based on this evidence and different *in silico* analysis including molecular fold predictions and dynamic simulations, the fusion activity of hantaviruses has been associated with Gc, together with overall structural characteristics similar to those of class II fusion proteins [29] (Figure 3). For other members of the *Bunyaviridae* family, the fusion activity has also been associated with Gc [28,30]. As mentioned above, the recent resolution at a 1.9 Å of the crystal structure of RVFV Gc confirmed that this phlebovirus protein has a remarkable similarity to the E1 and E class II fusion proteins of alphaviruses and flaviviruses, respectively [27].

To initiate membrane fusion, a specific trigger must activate the viral fusion protein; this can include separately or combined factors such as low-pH, interactions with receptors, contact with a target membrane, and disulfide shuffling, among others (reviewed in [58]). For hantaviruses, low pH is necessary for membrane fusion (Figure 2, step 4). Using cell-cell fusion assays of cells expressing Gn and Gc, a pH of 6.3 has been determined for HTNV while a pH of 5.8 has been identified for ANDV [31,62]. Additional host-cell derived triggers may be required for hantavirus Gc fusion activation; one that has been proposed is a thiol-isothiomerase [63].

For low-pH activated fusion proteins, the protonation of specific histidine residues (pKa ~6.5) serves as “sensor” of low pH exposition [64,65,66,67]. The Gc protein of hantaviruses contains a rather high number of conserved histidines and none of them has yet been associated with a role as trigger of the fusion activity. For Gc of the phlebovirus RVFV, substitutions of histidine residues H857A, H778A and H1087A were sufficient to abrogate or impair virus entry. Moreover, mutant H857A proved to be insensitive to acid-activated homomultimerization [68]. Such homo-multimerization arrangements have been described for many currently characterized viral fusion proteins [69,70]; however, a low pH-induced multimeric rearrangement has not yet been described for the Gc fusion protein of hantaviruses. Interestingly, the treatment of TULV particles at low pH decreases their infectivity by 100-fold, indicating that irreversible changes seem to occur in the fusion protein [71].

After fusion protein activation, fusion peptides must insert into the target membrane, contributing to their destabilization [72,73]. Based on the crystal structure of RVFV Gc, it has been proposed that residues W821, F826, and V828, contained in the fusion loop region at the tip of the protein, initiate interaction with target membranes [27]. For hantaviruses, it has been experimentally shown that the conserved fusion loop candidate includes essential residues for fusion activity [31] (see Figure 3). Specifically, wt Gn and mutant Gc including substitutions within the fusion loop candidate region were tested for their cell-cell fusion activity and infectivity upon incorporation onto lentivirus particles. Substitutions of aromatic and polar Gc residues W766A, W766F, N769A abrogated cell-cell fusion and infection [31]. Interestingly, it has been shown that peptides spanning this region interact with artificial membranes, preferably enriched with cholesterol and sphingomyelin, typically present in lipid rafts [29]. Based on these observations, it is likely that these residues play a role in the interaction with membranes; W766 may drive the insertion of the fusion loop into the target membrane while polar residues may stabilize the loop structure, or alternatively, may establish ionic interactions with the membrane head groups [31].

During recent years, exciting studies have allowed the characterization of several late intermediate steps for viral fusion proteins of different structural classes, before they reach their final hairpin conformation [74,75,76]. Among class II fusion proteins, the driving force for the fold-back reaction seems to be given by domain III, which moves about 37 Å towards the fusion peptide [70]. During this translocation, the more C-terminal stem region zippers up against the core-trimer [74,75]. Whether the fusion proteins of hantaviruses and other bunyaviruses contain such regions, and whether they may follow a similar strategy to reach a post-fusion conformation, remain as questions that warrant future research.

## 4. Biogenesis of the Viral Glycoproteins

The hantavirus Gn and Gc glycoproteins are encoded within a unique ORF by the genomic M segment as a precursor polypeptide of 1,133 to 1,148 residues, termed glycoprotein precursor (GPC, see Figure 3) [77]. Sequence-based predictions indicate the presence of four membrane-spanning domains, two of which are thought to serve as signal peptides for translocation into the lumen of the ER, while the other two are thought to serve as transmembrane domains for anchoring each protein to the phospholipid membrane.

The mature HTNV Gn protein has been shown to start at residue 18 after its first AUG codon [77], according to the typical processing of hydrophobic leader sequences in secretory and type-I transmembrane proteins [78]. On the other hand, HTNV Gc is thought to start at residue 649 after the third membrane-spanning sequence [77,79]. This notion has been further supported by mutagenesis studies of the highly conserved sequence motif WAASA (HTNV GPC residues 644–648). When residue substitutions were introduced into this motif, the cleavage of GPC was abrogated, giving rise to an unprocessed ~120 kDa precursor, evidencing against a secondary cleavage site in GPC [80]. It is thought that the conserved WAASA motif may be cleaved by a cellular signal peptidase complex [80], giving rise to the Gn and Gc proteins, derived from the N-terminal and C-terminal of GPC, respectively. Once proteolytic processing is completed, Gn and Gc are believed to each be anchored by their transmembrane domain to the phospholipid membrane and to direct its N-terminal domain towards the lumen of the secretory pathway and subsequently towards the extracellular space [79]. Processing of glycoproteins and post-translational modifications are depicted in a scheme in Figure 3.

In order to exit from the ER, heteromultimerization of Gn and Gc glycoproteins seems to be a pre-requisite [81,82,83]. The Gn and Gc of other bunyaviruses also seem to form heteromeric associations in the ER before being transported to the Golgi apparatus [79,84]. For further hantavirus glycoprotein maturation, their modification by N-linked glycosylations is essential [77,83]. Using site-directed mutagenesis studies, four glycosylation sites were identified on HTNV Gn (N134, N235, N347 and N399) and one on HTNV Gc (N928) [85]. Among these sites, the N-glycosylation signal sequence motif NxS/T is not present at position N235 of most hantaviruses. N-glycans of HTNV Gn and Gc glycoproteins are endoglycosidase-H sensitive, indicating that they are of high-mannose type [83,85] (Figure 3). Further, putative O-glycosylations of Gn and Gc also have been identified [86,87]. Furthermore, a serine at position 1124 of the HTNV Gc protein has been previously proposed as a potential site of palmitoylation [88].

As described, hantaviruses expose spikes that likely consist of four Gn and four Gc proteins [16,17]. In this sense, it seems probable that these multimeric associations are already assembled in the ER, upon glycoprotein synthesis and folding, leading to the trafficking of glycoprotein heteromultimers to viral assembly sites. Lateral interactions among these complexes may occur once a high local concentration of glycoproteins is reached.

## 5. Roles of the Glycoproteins during Virus Cell Exit

The assembly of the structural components of enveloped viruses occurs generally at specific sites, mostly at microdomains on the plasma membrane or in the internal compartments of the cell [5,6]. In the latter case, viruses must egress the cell as cargo down the secretory pathway via exocytosis. For hantaviruses, it has been assumed that their assembly occurs at the Golgi apparatus, similar to other members of the *Bunyaviridae* family [79,89,90,91] (Figure 2, step 6a). This has been associated with the retention of the viral glycoproteins at this cellular compartment [81,92]. However, ANDV, HTNV, and SNV glycoproteins have also been detected on the plasma membrane at later timer points, post-transfection or post-infection [31,60,92]. In this context, it is not surprising that the membrane budding of American hantaviruses, such as SNV and BCCV, has also been observed from the plasma membrane [15,93] (Figure 2, step 6b). A report on a new hantavirus isolate suggested virus maturation at multiple sites in Vero E6 cells, including the ER [94]. Yet specific hantavirus assembly sites remain to be characterized more specifically *in vitro* and *in vivo* in the future.

### 5.1. Interactions of the Glycoproteins with Internal Viral Components

The assembly of enveloped viruses involves in general the association of three major structural components at a membrane; envelope glycoproteins, matrix proteins and ribonucleocapsids containing the viral genome. Among these components, matrix proteins usually connect these interactions [6,95]. Enveloped viruses such as togaviruses and bunyaviruses lack matrix proteins; however, it has been reported that the cytoplasmic tails of both the Gn (~150 aa) and Gc (~9–26 residues) of hantaviruses and other bunyaviruses can interact directly with the N protein or with ribonucleocapsid complexes [96,97,98,99,100] (Figure 3, RNC-BS). Hence, these endodomains may act as matrix surrogates, establishing communication between the external and the internal components of the virus particles [96,97,98,99,100,101]. Interestingly, the interaction between N and Gn-CT depends on a properly folded dual zinc finger domain [97] previously described by Estrada and colleagues (Figure 3) [24]. Zinc fingers are common protein motifs that are generally involved in the recognition of specific proteins or that bind nucleic acids [102,103,104]. They are typically present in viral proteins that interact with nucleic acid, such as nucleocapsid proteins [105,106,107]. However, the dual ββα-zinc finger domain located in the hantavirus Gn-CT does not seem to interact directly with RNA [24]. Instead, it has been reported that the C-terminal part of the Gn-CT, downstream of the dual zinc finger domain, binds RNA unspecifically [108]. For other bunyaviruses, the Gn-CT of CCHFV has been shown to interact with RNA, directly through the dual zinc finger domain [26]. In the case of RVFV, a zinc finger fold in the Gn-CT is not likely [26]; however, Gn-CT seems to directly interact with the nucleoprotein and the viral RdRp [109]. All the interactions that the bunyavirus glycoprotein CTs establish with viral internal components such as RNA, nucleoprotein, and RdRp may help during the packaging of the viral genome. How these viruses coordinate the packaging of ribonucleocapsids that contain the three different viral genomic segments continues to be a mystery among all viruses with segmented genomes.

### 5.2. Roles of the Glycoproteins during Virus Budding

Once all structural components interact with each other, virus budding requires membrane evagination and scission to allow the separation of the virus from the membrane, thus leading to the release of a new virus particle. In general, this budding process requires a driving force that induces membrane curvature, which is usually achieved by the interaction of viral proteins with membrane lipids [6]. Virus budding can be driven by at least three different interactions between viral proteins: (i) by interactions of the surface glycoproteins; (ii) by interactions of internal viral proteins such as matrix proteins; and (iii) by the concerted interaction of surface glycoproteins together with internal proteins [5]. As a matrix protein is absent among bunyaviruses, only alternatives (i) and (iii) are possible budding impulses for these viruses.

To study virus budding, individual viral proteins can be expressed in cells and supernatants can be subsequently analyzed for the release of virus structures known as virus-like particles (VLPs) [6]. A recent report described for the first time that the Gn and Gc glycoproteins of ANDV and PUUV self-assemble into VLPs [110], confirming that these glycoproteins can indeed provide the driving force for hantavirus budding in the absence of other viral components. Hence, although hantaviruses expose only locally ordered spikes organized in patches on their envelope, the contacts between these spikes seem to be enough to trigger membrane curvature. For HTNV, the formation of VLPs has been reported to occur when the glycoproteins are expressed together with the viral nucleoprotein [111,112]. Based on the observed cryo-ET density of HTNV spikes [16], which lack the lateral spike interactions with peripheral stalks observed in the TULV map [17], it would be interesting to test in the future whether HTNV glycoproteins self-assemble into VLPs in the absence of the nucleoprotein.

Within the *Bunyaviridae* family, the formation of VLPs has been also reported for phleboviruses [113,114]. Among them, the expression of the Gn and Gc envelope glycoproteins of phleboviruses is sufficient to allow the generation and release of VLPs, hence indicating that phlebovirus budding seems to be mainly driven by their envelope glycoproteins. This is consistent with their icosahedral symmetry which establishes regularly spaced contacts among the surface spikes.

As described above, the hantavirus surface spikes form locally ordered patches with a four-fold symmetry [16,17]. In addition to the local four-fold symmetry of the hantavirus spikes, the TULV cryo-ET map also exposed membrane patches on the particle surface [17]. As square-shaped surface spikes of four-fold symmetry are not compatible with a polyhedral symmetry, uncovered membrane patches seem to be necessary to allow geometric consistency and hence particle closing.

In order to achieve the pinching-off reaction of budding virus particles, many enveloped viruses recruit the cellular ESCRT (Endosomal Sorting Complexes Required for Transport) machinery that mediates the formation of multivesicular bodies. These cellular complexes are recruited by ubiquitination of residues within proteins present at the membrane of internal compartments (reviewed in [5]). In a recent review, a question was formulated as to whether the highly conserved Y619, which forms the ITAM motif YxxL within Gn-CT (Figure 3), may function as a late domain for the recruitment of cellular ESCRT complexes [101]. This hypothesis is particularly attractive, since the ubiquitination of this ITAM motif has been shown to be involved in New York-1 virus Gn ubiquitination and subsequent degradation via proteasome [115]. The downregulation of Gn through the ubiquitination of these tyrosines or other Gn-CT residues has also been shown for other hantaviruses, whereby they may escape host cell immune responses [115,116,117]. Taken together, the recent progress in the characterization of the Gn-CT points towards its role as a multifunctional domain; it probably participates in different steps of the viral replicative cycle, such as glycoprotein down regulation through different degradation pathways, interaction with nucleoproteins and RNA during virus assembly and may even promote membrane scission. In contrast, the shorter ANDV Gc-CT does not seem to be involved in virus particle assembly and egress [110].

## 6. Glycoprotein Arrangements on Hantavirus Particles

Structural perspectives of virus structures during early and late events of virus replication have allowed for a discerning of the dynamic transitions of envelope proteins on virus particles; although these proteins form part of the defined spike complexes of mature particles, during cell entry and exit they undergo re-arrangements. Structural plasticity allows them to accomplish their functions during virus assembly, budding, maturation, receptor interaction and membrane fusion as well as to hide epitopes from the neutralizing antibodies of the host cell immune system [118,119,120,121]. Insights into envelope protein arrangements and transitions have allowed the drawing of a detailed picture of the mechanism behind virus cell entry and exit steps.

In this sense, the different spike electron densities observed between TULV and HTNV cryo-ET maps [16,17] (see Figure 1) could correspond to dissimilarities between these viruses under equivalent conditions or may have been produced by different technical proceedings. In the latter case, preparation of the sample may have caused a spike conformation that does not occur on native hantaviruses, or alternatively, it may have captured different spike transitions during the virus life cycle. To achieve a deeper understanding of the entry and exit mechanisms of hantaviruses, it would be of huge value to obtain structural perspectives of how Gn and Gc are arranged on the virus surface during some of these steps.

As described above, the particularity of the spike density found in the TULV cryo-ET map [17] allows one to distinguish two units; one corresponding to the outermost exposed tetrameric lobes, which form the local four-fold symmetry, and another unit, corresponding to peripheral stalks. Data from different authors point towards the hypothesis that the tetrameric lobes of TULV contain Gn tetramers that may be interconnected by Gc dimers (Figure 1B):

(i) During Gn and Gc biogenesis, the glycoproteins seem to require hetero-oligomerization in order to exit the ER (see above). Before entering the secretory pathway, fusion proteins must shield their fusion peptides; this is usually achieved through intermolecular interactions [58]. In the case of class II fusion proteins this interaction occurs with a companion protein [118]. Hence, to hide the fusion loop peptide of Gc, it is likely that this region interacts with Gn; alternatively it may interact with another Gc molecule in a juxtaposed position. In this regard, it is interesting to note that the residue substitution D121N, located in the fusion loop candidate of ANDV Gc, abrogates its incorporation onto lentivirus particles [31]. It can be speculated that this substitution may impede the intermolecular interactions of the fusion loop with Gn or Gc, which may be involved in the Gn/Gc quaternary assembly. In the context of the spike arrangements, it is therefore more likely that Gc forms part of the more buried, peripheral stalks. However, the volume of the electronic density occupied by the peripheral stalks seems to be too small to accommodate a class II fusion protein. Hence, if hantavirus Gc is arranged in a “class II fold”, then it must occupy also part of the electron density corresponding to the tetrameric lobes
(ii) It has been known for a long time that Gn forms SDS-resistant oligomers, even under reducing conditions [71,83,84,85]. Using reducing agents and cross-linking techniques, Hepojoki and colleagues [71] demonstrated that one of the Gn oligomeric arrangements corresponds to a tetramer. If, the most exposed tetrameric lobes on the virus surface, thus correspond to Gn tetramers, then Gn may in turn correspond to the protein that establishes the first interactions with cell surface molecules during virus attachment to the c

The notion that TULV particle spikes may correspond to Gn tetramers interconnected by Gc dimers has also been proposed by others [122]. In the case of HTNV Cryo-ET, each tetrameric lobe contains a high stalk density beneath and no interconnecting units can be observed [16]. Bearing in mind that the tetrameric spike complexes probably correspond to four Gn and four Gc subunits, it can be hypothesized that each monomeric subunit of the tetrameric HTNV spike density may correspond to a Gn/Gc heterodimer (Figure 1B). In the future, it will be exciting to experimentally test the Gn and Gc arrangements within hantavirus spikes. To this end, the recently developed VLP system for ANDV and PUUV hantaviruses is currently being used to assay different glycoprotein mutants for VLP self-assembly and multimerization. How the square-shaped tetrameric spikes, containing most probably four Gn and four Gc proteins, rearrange upon Gc activation during cell entry will be another interesting area of study. Currently characterized fusion proteins of all three classes are known to form homotrimeric associations upon activation. Whether the hantavirus Gc homotrimerizes upon activation and how such rearrangements could be achieved based on the local, tetrameric symmetry, remains another unexplored field.

## 7. Conclusions

The pieces describing the steps of the hantavirus life cycle are just starting to be characterized and brought together as a complex puzzle. An amazing diversity in the structures of viral-spike complexes has been found among three genera of the *Bunyaviridae* family. Despite this information, studies on the assembly and budding mechanisms of these viruses are still in their infancy. Recently, more novel insights have been gained regarding the entry processes used by these viruses to invade target cells. Hopefully, more pieces of the puzzle will be uncovered in the near future, such as the molecular structures of the hantavirus glycoprotein ectodomains and the understanding of how the glycoproteins are involved in the different coordinated and successive steps during the viral life cycle. Finally, these insights may allow for the rational design of effective countermeasures against these highly pathogenic viruses.
